# The relationship between trust in mass media and the healthcare system and individual health: evidence from the AsiaBarometer Survey

**DOI:** 10.1186/1741-7015-7-4

**Published:** 2009-01-22

**Authors:** Yasuharu Tokuda, Seiji Fujii, Masamine Jimba, Takashi Inoguchi

**Affiliations:** 1 Center for Clinical Epidemiology, St. Luke's Life Science Institute, St. Luke's International Hospital, 9-1 Akashi-cho, Chuo City, Tokyo 104-8560, Japan; 2 Graduate School of Political Science, Chuo University, Tokyo, Japan; 3 Department of International Community Health, Graduate School of Medicine, The University of Tokyo, Tokyo, Japan

## Abstract

**Background:**

Vertical and horizontal trust, as dimensions of social capital, may be important determinants of health. As mass media campaigns have been used extensively to promote healthy lifestyles and convey health-related information, high levels of individual trust in the media may facilitate the success of such campaigns and, hence, have a positive influence on health. However, few studies have investigated the relationship between trust levels in mass media, an aspect of vertical trust, and health.

**Methods:**

Based on cross-sectional data of the general population from the AsiaBarometer Survey (2003–2006), we analyzed the relationship between self-rated health and trust in mass media, using a multilevel logistic model, adjusted for age, gender, marital status, income, education, occupation, horizontal trust, and trust in the healthcare system.

**Results:**

In a total of 39082 participants (mean age 38; 49% male), 26808 (69%) were classified as in good health. By the levels of trust in mass media, there were 6399 (16%) who reported that they trust a lot, 16327 (42%) reporting trust to a degree, 9838 (25%) who do not really trust, 3307 (9%) who do not trust at all, and 191 (0.5%) who have not thought about it. In the multilevel model, trust in mass media was associated with good health (do not trust at all as the base group): the odds ratios (OR) of 1.16 (95% confidence interval (CI) = 1.05–1.27) for do not really trust; OR of 1.35 (95% CI = 1.23–1.49) for trust to a degree, and 1.57 (95% CI = 1.36–1.81) for trust a lot. Horizontal trust and trust in the healthcare system were also associated with health.

**Conclusion:**

Vertical trust in mass media is associated with better health in Asian people. Since mass media is likely an important arena for public health, media trust should be enhanced to make people healthier.

## Background

Social capital has developed as a concept indicating the quantity and quality of social interactions in the community and has emerged recently as an important determinant of health [1]. A society with high levels of social capital has high social participation among its citizens, high interpersonal trust, and high levels of institutional or organizational trust [2,3]. Studies suggest that societies and individuals with higher social capital have positive effects on various aspects of physical and psychological health among individuals in those societies [4,5]. Social capital is considered to promote health through mechanisms including effective reciprocal support, mutual respect, better access to local services, social control of deviant behavior and violence, and enhanced transmission of health information and healthy behavior [6].

Although social capital has been assessed as social participation or social trust [3], recent studies have suggested that a society with high social participation but with low social trust is associated with high-risk adverse behaviors to health [7-10]. Trust has emerged recently as the central means of achieving cooperation in inter-organizational and inter-individual relationships and promoting the accumulation of social capital [3,11].

Social trust reflects the expectation that an individual or institution will act competently, fairly, openly, and with concern [12], and can be divided into horizontal (interpersonal) trust and vertical (institutional) trust [3]. Horizontal trust flows across and among ordinary people. Vertical trust flows upward from people to public institutions in a society [13]. Development of the capacity to trust others is an essential element for successful social adjustment [14], and is considered an important predictor of health and psychological well-being [15,16].

Persons with high vertical trust consider public institutions or organizations as trustful social resources and the levels of this vertical trust may vary between societies with the level of social connectedness [17]. For instance, the healthcare system is one of the important institutions in which people may feel different levels of trust. A higher vertical trust in the healthcare system has been shown to be associated with better self-rated health [17]. Patients with high trust in the healthcare system are likely to gain access to healthcare services, provide important medical information to healthcare providers, and may be better at following advice and completing prescriptions.

However, little is known about the nature or role of vertical trust in terms of health determinants between other institutions and individuals in society. In addition to the healthcare system, mass media is also considered one of the most important public institutions, and may have a considerable effect on public health through the levels of trust the people have in this institution [13], and vertical trust in mass media may be an important determinant of health.

Mass media may function well with respect to improving health, along with relevant aspects of trust. A potential pathway from high trust in mass media to better health is increased acceptance of health-related messages and the resultant dissemination of good behavior related to health throughout communities. For instance, a recent study has shown that improvements in exercise and diet mediated by community-level projects are associated with better mental health [18]. The authors of the study on the New Deal for Communities in the UK suggest that better mental health and health-related behavior occur through increasing community cohesion and social capital more widely in the neighborhood, beyond people involved directly in lifestyle interventions [18].

In addition, a recent study has shown that public health agencies, using their communication and marketing resources effectively to support people in making healthful decisions and to foster health-promoting environments, have considerable opportunity to advance public health [19]. Thus, those with high trust in both the healthcare system and mass media may be more likely to receive these positive, and possibly synergistic, effects on health.

Furthermore, the links between vertical and horizontal trust are well founded and are positively correlated in an amplifying cycle [20]. Indeed, a recent study has supported the trust propagation cycle, in which there are two types of vertical trust: vertical trust in representative institutions (input vertical trust) and trust arising from experience of the services provided (directly or indirectly) by such institutions (output vertical trust) [20]. Satisfaction with community services promotes vertical trust, as well as horizontal trust, and a trust cycle propagates trust within a community [20]. Thus, those with output vertical trust in mass media may be more likely to have higher trust in other institutions and horizontal trust, which can in turn lead to better health.

Despite the importance of examining the relationships between vertical trust in mass media, few studies have addressed these issues. Therefore, in this study, we aim at evaluating the association between distrust in mass media and poor health among Asians, using data from the AsiaBarometer Survey, comprising trans-national and multidimensional surveys conducted throughout Asia.

## Methods

### Study participants

We used data from the AsiaBarometer Survey (2003–2006), which included information on individuals from 29 Asian countries on a vast range of subjects [21]. The countries included in our analysis were Afghanistan, Bangladesh, Bhutan, Brunei, Cambodia, China, Hong Kong, India, Indonesia, Japan, Kazakhstan, Kyrgyzstan, Laos, Malaysia, the Maldives, Mongolia, Myanmar, Nepal, Pakistan, Singapore, South Korea, Sri Lanka, Taiwan, Tajikistan, Thailand, the Philippines, Turkmenistan, Uzbekistan, and Vietnam. For the purpose of the study, Hong Kong and Taiwan were considered independent countries, in view of their socioeconomic characteristics. Prior ethics committee approval was obtained from the Chuo University. We received written informed consent from the survey participants.

### Data collection

We used face-to-face interviews to administer structured questionnaires. The detailed content of the questionnaires has been published previously [21]. Data collection included demographics, marital status, socioeconomic factors (income, education, and occupation), self-rated health, interpersonal trust, and trust in the healthcare system and mass media, as well as information on political, environmental, and daily-life issues that were related to the AsiaBarometer Survey.

The individual-level independent variables included gender, age (range between 20 and 69 years), marital status, religious belief, income, education, employment, and individual-level social trust. Age was categorized into five groups of 20–29, 30–39, 40–49, 50–59, and 60–69 years old. Categories of marital status included single, married, divorced/separated, or widowed.

Annual household income was used as an income variable in this study. Categories of the income groups included low, middle, and high, based on the income distribution of each country (see Appendix A, in Additional file 1). For educational achievement, we also used three categories (low, middle, and high) based on the distribution of educational achievement in each country (see Appendix B, in additional file 1). For occupational status, six categorical classes were used: self-employed, employed, unemployed, retired, homemaker, and student. The self-employed group included: self-employed in agriculture, forestry or fisheries; business owner in mining or manufacturing industry of an organization with up to 30 employees; vendor or street trader; business owner or manager of an organization; and self-employed professional. The employed group included senior manager, employed professional or specialist, clerical worker, sales, manual worker, driver, and "other" worker.

In this study, self-rated health was defined as the individual's personal satisfaction with their overall health. In the survey, we asked "Please tell me how satisfied or dissatisfied you are with your health? Would you say you are very satisfied, somewhat satisfied, neither satisfied nor dissatisfied, somewhat dissatisfied, or very dissatisfied with your health?". These categories were collapsed to form a dichotomous outcome of self-rated health: poor health (1) for very dissatisfied, somewhat dissatisfied, or neither satisfied nor dissatisfied; and good health (0) for very satisfied, or somewhat satisfied.

Horizontal trust, a dimension of cognitive social capital, was measured by a composite index constructed from a factor (principal component) score of three questionnaire items related to general trust, interpersonal trust, and mutual help. The general trust question was, "Would you say that most people can be trusted or that you can't be too careful in dealing with people?". The question for interpersonal trust in merit-based utility was, "Would you say that most of the time people try to be helpful or that they are mostly looking out for themselves?". The question for mutual help was, "If you saw somebody on the street looking lost, would you stop to help?". For the last question, the responses were: "I would always stop to help", "I would help if nobody else did", and "It is highly likely I wouldn't stop to help". These questions have been widely used in previous studies to measure cognitive social trust [2,5,22,23]. Factor analysis of these items provided a one-factor solution with an eigenvalue of 1.4. All items were loaded above 0.4 and no other factors exceeded unity. The individual scores were calculated using the regression equation with the factor loadings, and a higher score indicated lower trust. The scores were then standardized (mean 0; standard deviation 1). Before being included into the multivariable multilevel model, the scores were further collapsed to form a dichotomized variable: low social trust (0) for the values less than 0 and high social trust (1) for the values of 0 or more.

Trust in institutions (vertical trust) is an item that reflects the participant's trust in the healthcare system and in mass media (specified as newspapers and television). The item "Please indicate to what extent you trust the following institutions to operate in the best interests of society" offered the alternatives (a) the healthcare system and (b) mass media, with the six alternative responses: (1) "Trust a lot"; (2) "Trust to a degree"; (3) "Don't really trust"; (4) "Don't trust at all"; (5) "Haven't thought about it"; and (6) "I don't know".

### Statistical analysis

Descriptive statistics were calculated and presented as the mean with standard deviation or the count number in proportion to the overall sample population where appropriate. Bivariate correlation analyses were conducted among the trust variables using Pearson's correlation coefficients.

We used the multilevel (mixed-effects) logistic regression model to analyze the relationship of individual characteristics to self-rated health by considering individuals nested in each country, as data structures in the Asia Barometer Survey were hierarchical multilevels (level 1, individual; level 2, country). The data provide information on individuals, while the individuals are also grouped in their countries. Analyzing hierarchical data at the individual level by conventional regression models does not meet the assumption of independence of observations. When ignoring the nesting of individuals in countries, the estimated standard errors would be smaller, thus inflating the risk of Type I errors [24]. The mixed-effects model can be used to analyze hierarchical data [24], and is used widely in social and epidemiological research. The random-effects covariance matrix was set to an unstructured form and we utilized three trust measures (horizontal trust, trust in the healthcare, and trust in mass media) as the random-effects parameters in the model. Variances and their standard errors were estimated for these random-effects parameters.

The model was constructed to evaluate the relations of trust in the healthcare system and mass media to self-rated health, adjusted for age, gender, marital status, income, education, occupation, and horizontal trust. We constructed a total of six models, including only baseline sociodemographic variables (base), such as age, gender, marital status, income, education, and occupation (Model 1), base plus horizontal trust (Model 2), base plus trust in the healthcare system (Model 3), base plus trust in mass media (Model 4), base minus income and education plus horizontal trust, trust in the healthcare system and trust in mass media (Model 5), and base plus horizontal trust, trust in the healthcare system and trust in mass media (Model 6; full model). Model 5 was constructed by eliminating income and education from the full model for examining the possible endogeneity to health of income and education.

No interaction terms were included in the model. To check the robustness of the model, we also conducted the logistic regression analysis including country fixed effects as well as the ordered probit model analysis using original dependent variable (self-rated health). The odds ratios (ORs) along with 95% confidence interval (CIs) were estimated in each variable for poor health. An OR value greater than one indicates greater effects that were positively related to poor health. All statistical analyses were performed using STATA 10.0 (College Station, TX, USA). Two-tailed *P*-values less than 0.05 were considered statistically significant.

## Results

Table 1 presents the descriptive statistics of the study participants. The sample population was split almost evenly between women and men. The mean age was 37.8 years (standard deviation (SD) = 11.9). The majority of participants were married (72.4%). The three levels of both income and education were distributed almost evenly. In terms of job status, the majority were employed: employed (48.2%) and self-employed (16.5%).

**Table 1:** Sociodemographics of all participants (N = 39082)

Characteristic	No.	%
Demographics		
Gender		
* Women	19800	50.7
Men	19282	49.3
Age, yr		
* 20–29	11413	29.2
30–39	11128	28.5
40–49	9147	23.4
50–59	5784	14.8
60–69	1610	4.1
Marital status		
* Married/partnered	28278	72.4
Others	10772	27.6
NA	32	0.1

Socioeconomic Status		
Income		
* High	12420	31.8
Mid	12219	31.3
Low	12426	31.8
NA	2017	5.2
Education		
* High	11861	30.3
Mid	14549	37.2
Low	12518	32.0
NA	154	0.4
Employment		
* Self-employed	6467	16.5
Employed	18843	48.2
Unemployed	13681	35.0
NA	91	0.2

NA = data not available.

* Reference categories used for subsequent regression analyses.

In terms of self-rated health, 68.6% considered themselves to be in good health, while 30.9% were in poor health. More than half (55.4%) of the participants were classified as having low horizontal trust (Table 2). For the questionnaire involving trust in the healthcare system and mass media, the majority (64.1%) of participants were classified as having trust ("trust a lot" and "trust to a degree") in the healthcare system, and similarly, 58.1% of the participants were classified as having trust in mass media.

**Table 2:** Levels of Horizontal Trust and Trust in the Healthcare System and in Mass Media by Health Status

Characteristic	All participants (N = 39082)		Good health (n = 26808)		Poor health (n = 12080)	
	No.	%	No.	%	No.	%
Horizontal trust						
High	14450	37.0	10170	37.9	4206	34.8
* Low	21642	55.4	14637	54.6	6918	57.3
NA	2990	7.7	2001	7.5	956	7.9

Trust in the healthcare system						
Trust a lot	7568	19.4	5971	22.3	1551	12.8
Trust to a degree	17475	44.7	12364	46.1	5062	41.9
Don't really trust	7934	20.3	4732	17.7	3161	26.2
* Don't trust at all	2344	6.0	1234	4.6	1086	9.0
Haven't thought about it	71	0.2	48	0.2	23	0.2
NA	3690	9.4	2459	9.2	1197	9.9

Trust in mass media						
Trust a lot	6399	16.4	4801	17.9	1554	12.9
Trust to a degree	16327	41.8	11716	43.7	4571	37.8
Don't really trust	9838	25.2	6401	23.9	3406	28.2
* Don't trust at all	3307	8.5	1948	7.3	1346	11.1
Haven't thought about it	191	0.5	119	0.4	72	0.6
NA	3020	2.6	1823	6.8	1131	9.4

NA = data not available. * Reference categories used for subsequent regression analyses.

For horizontal trust, 37.9% of the participants with good health and 34.8% with poor health had high trust (*P *< 0.001). For trust in the healthcare system, 22.3% of the participants with good health and 12.8% with poor health reported as "having trust a lot" (*P *< 0.001). In addition, for trust in mass media, 17.9% of the participants with good health and 12.9% with poor health reported as "having trust a lot" (*P *< 0.001).

The correlation coefficient between trust in the healthcare system and trust in mass media was 0.3434 (*P *< 0.001). The correlation coefficients between horizontal trust and trust in the healthcare system and between horizontal trust and trust in mass media were 0.0159 and 0.0160, respectively (*P *< 0.001 for both).

Table 3 presents the mean scores and standard deviations of health and trust for each of the 29 countries. By construction, the horizontal trust score of all participants was centered at 0 with a standard deviation of 1. In terms of self-rated health, people in Brunei also reported the highest level, followed by those in Bhutan and Indonesia. People in Turkmenistan reported the lowest level of health, followed by those in Cambodia and Mongolia.

**Table 3:** Health, Horizontal Trust, and Trust in the Healthcare System and in Mass Media in 29 Asian countries

Country	No.	Health *		Trust					
				Horizontal **		Healthcare system ***		Mass media ***	
		mean	SD	mean	SD	mean	SD	mean	SD
Afghanistan	874	4.11	0.98	0.25	1.00	1.96	0.81	1.83	0.92
Bangladesh	1008	3.87	1.04	-0.18	0.82	2.05	0.79	2.14	0.86
Bhutan	801	4.38	0.81	0.01	0.97	2.38	0.67	2.09	0.71
Brunei	804	4.62	0.57	0.21	0.94	2.71	0.49	2.20	0.68
Cambodia	812	3.29	1.05	-0.64	0.65	1.86	0.80	1.91	0.75
China	3800	3.71	0.95	0.54	1.02	1.54	0.75	1.48	0.79
Hong Kong	1000	3.57	0.71	0.06	1.06	1.65	0.73	0.95	0.72
India	2060	4.25	0.94	-0.08	0.97	1.84	0.82	2.12	0.84
Indonesia	825	4.35	0.84	0.07	0.90	2.27	0.67	2.06	0.68
Japan	2685	3.66	0.98	-0.01	1.01	1.56	0.67	1.16	0.68
Kazakhstan	800	3.47	1.16	-0.41	0.80	1.72	0.81	1.66	0.80
Kyrgyzstan	800	3.57	1.27	-0.32	0.73	1.66	0.90	1.72	0.83
South Korea	2642	3.55	0.91	0.46	1.02	1.41	0.69	1.33	0.74
Laos	800	3.92	0.98	-0.33	0.86	2.16	0.65	1.82	0.72
Malaysia	1600	4.22	0.75	-0.28	0.92	2.42	0.60	1.78	0.72
Maldives	821	4.34	0.87	0.55	0.97	2.69	0.56	2.67	0.75
Mongolia	800	3.42	1.09	-0.18	0.88	1.84	0.78	1.73	0.76
Myanmar	1600	3.78	1.12	-0.17	0.84	NA		1.94	0.70
Nepal	800	3.81	0.78	-0.24	0.79	1.74	0.70	2.11	0.64
Pakistan	1086	3.51	1.02	0.49	1.01	1.51	0.86	1.63	0.87
the Philippines	800	4.21	0.84	-0.50	0.80	2.17	0.68	2.16	0.70
Singapore	1838	4.06	0.75	0.10	1.02	2.21	0.57	1.74	0.69
Sri Lanka	1613	4.13	0.86	-0.32	0.93	1.92	0.72	1.59	0.84
Taiwan	1006	3.62	0.84	0.09	1.13	1.67	0.72	1.05	0.82
Tajikistan	800	3.85	1.04	-0.07	0.97	1.23	0.91	1.71	0.89
Thailand	1600	3.82	1.07	-0.33	0.89	2.17	0.70	1.80	0.70
Turkmenistan	800	3.07	1.56	0.02	1.31	1.55	1.18	2.02	1.01
Uzbekistan	1600	3.43	1.15	-0.25	0.94	1.32	0.89	1.11	0.92
Vietnam	2607	3.56	0.95	0.11	0.94	2.05	0.75	2.16	0.74

Total	39082	3.81	1.02	0.00	1.00	1.86	0.83	1.72	0.86

* Based on 5-point Likert scale from very dissatisfied with health (1) to very satisfied with health (5).

** Based on 1-factor analysis from the three questionnaires. The greater value indicates the higher trust.

*** Based on 4-point Likert scale from "Don't trust at all" (0) to "Trust a lot" (3). NA = data not available. SD = standard deviation.

People in the Maldives reported the highest level of trust in mass media, followed by those in Brunei and the Philippines, while people in Hong Kong reported the lowest level of trust in mass media, followed by those in Taiwan and Uzbekistan. In addition, for the horizontal trust score, people in the Maldives reported the greatest level of trust, followed by those in China and Pakistan. People in Cambodia reported the lowest level of trust, followed by those in the Philippines and Kazakhstan. Lastly, people in Brunei reported the highest level of trust in the healthcare system, followed by those in the Maldives and Malaysia, while people in Tajikistan reported the lowest level of trust in the healthcare system, followed by those in Uzbekistan and South Korea. Data for trust in the healthcare system in Myanmar was not available at the time of the survey.

Table 4 presents the results from six multilevel logistic regression models for good health, adjusted for age, gender, marital status, income, education, occupation, horizontal trust, and trust in the healthcare system and mass media. In Models 1, 2, 3, 4, and 6, the sociodemographic variables that were associated significantly with better health included women, younger age, marital status, high income, and high education (not mid education). Employment status was not associated with health in any of the models. Horizontal trust, trust in the healthcare system, and media trust were all significantly associated with good health in Models 2–6.

**Table 4:** Estimated Odds Ratios from Multilevel Logistic Models (outcome of good health)

Variable	Model 1	Model 2	Model 3	Model 4	Model 5	Model 6 **
*Fixed parameters*						
Male gender	1.21 (1.15–1.27) *	1.22 (1.16–1.29) *	1.22 (1.15–1.28) *	1.21 (1.15–1.28) *	1.25 (1.18–1.32) *	1.23 (1.16–1.30) *
Age						
30–39 yr	0.73 (0.68–0.78) *	0.73 (0.68–0.79) *	0.73 (0.68–0.79) *	0.76 (0.70–0.81) *	0.71 (0.66–0.77) *	0.75 (0.69–0.81) *
40–49	0.59 (0.55–0.63) *	0.59 (0.55–0.64) *	0.60 (0.55–0.65) *	0.62 (0.57–0.67) *	0.57 (0.53–0.62) *	0.62 (0.57–0.67) *
50–59	0.45 (0.41–0.49) *	0.44 (0.41–0.48) *	0.46 (0.42–0.50) *	0.47 (0.43–0.51) *	0.42 (0.39–0.46) *	0.46 (0.42–0.50) *
60–69	0.41 (0.36–0.47) *	0.40 (0.35–0.45) *	0.42 (0.37–0.48) *	0.43 (0.37–0.49) *	0.36 (0.32–0.41) *	0.40 (0.35–0.46) *
Marital status						
Others	0.78 (0.74–0.83) *	0.80 (0.75–0.85) *	0.79 (0.74–0.84) *	0.82 (0.77–0.87) *	0.81 (0.76–0.86) *	0.81 (0.76–0.87) *
Income						
Mid	0.87 (0.82–0.93) *	0.87 (0.82–0.93) *	0.87 (0.82–0.93) *	0.88 (0.82–0.93) *		0.87 (0.81–0.93) *
Low	0.73 (0.69–0.77) *	0.73 (0.68–0.78) *	0.73 (0.69–0.78) *	0.73 (0.68–0.78) *		0.73 (0.68–0.78) *
Education						
Mid	1.00 (0.94–1.06)	1.01 (0.95–1.08)	0.99 (0.93–1.05)	1.00 (0.93–1.06)		1.00 (0.93–1.07)
Low	0.82 (0.76–0.88) *	0.82 (0.76–0.88) *	0.79 (0.74–0.86) *	0.82 (0.76–0.88) *		0.80 (0.74–0.87) *
Employment						
Employed	1.00 (0.94–1.07)	1.01 (0.94–1.08)	1.01 (0.94–1.09)	1.03 (0.96–1.11)	1.07 (0.99–1.15)	1.03 (0.95–1.11)
Unemployed	0.97 (0.90–1.05)	0.98 (0.91–1.06)	0.99 (0.92–1.08)	0.99 (0.92–1.08)	1.00 (0.92–1.09)	1.01 (0.93–1.10)

Horizontal trust						
High		1.29 (1.19–1.40) *			1.28 (1.17–1.39) *	1.27 (1.17–1.38) *
Trust in the healthcare system						
Don't really trust			1.32 (1.19–1.46) *		1.27 (1.13–1.43) *	1.29 (1.14–1.45) *
Trust to a degree			1.85 (1.64–2.08) *		1.72 (1.52–1.94) *	1.75 (1.54–1.99) *
Trust a lot			2.55 (2.18–2.97) *		2.27 (1.93–2.66) *	2.29 (1.95–2.68) *
Trust in mass media						
Don't really trust				1.25 (1.15–1.37) *	1.17 (1.07–1.28) *	1.16 (1.05–1.27) *
Trust to a degree				1.55 (1.42–1.69) *	1.34 (1.22–1.47) *	1.35 (1.23–1.49) *
Trust a lot				1.98 (1.73–2.27) *	1.56 (1.35–1.79) *	1.57 (1.36–1.81) *

*Random parameters*						
Between-country variation	0.22 (0.83)	0.22 (0.82)	0.18 (0.64)	0.20 (0.74)	0.17 (0.61)	0.18 (0.63)

Figures in parentheses are the 95% confidence intervals (except for between-country variation, for which each of the numbers corresponds to the standard error and the variance.

* Statistically significant at the 0.05 level.

** The regression model including country fixed effects showed the similar findings. In addition, the ordered probit model analysis using original dependent variable (self-rated health) produced the similar findings.

Based on the full model (Model 6), horizontal trust was associated significantly with good health, with an OR of 1.27 (95% CI = 1.17–1.38). For institutional trust ("don't trust at all" as the base group), trust in the healthcare system was associated significantly with good health, with ORs of 1.29 (95% CI = 1.14–1.45) for "don't really trust", 1.75 (95% CI = 1.54–1.99) for "trust to a degree", and, similarly, 2.29 (95% CI = 1.95–2.68) for "trust a lot". Overall, these results indicate a linear relationship between the levels of trust in the healthcare system and the ORs for good health (Model 3, 5, and 6 of Table 4). Similarly, trust in mass media was associated significantly with good health, with ORs of 1.16 (95% CI = 1.05–1.27) for "don't really trust", 1.35 (95% CI = 1.23–1.49) for "trust to a degree", and 1.57 (95% CI = 1.36–1.81) for "trust a lot". Again, these results indicate a linear relationship between the levels of trust in mass media and the ORs for good health (Models 4, 5, and 6 of Table 4). In addition to covariates in the full model, the regression model including country fixed effects showed similar findings and did not affect the results. Further, the ordered probit model analysis using the original dependent variable (self-rated health) produced the similar findings and did not affect the results.

## Discussion

The results of the current study suggest that trust in mass media is associated significantly with self-rated health. Slightly over 50% of the Asian participants reported that they "trust a lot" or "trust to a degree" in mass media. Trust in mass media remains associated significantly with health in multilevel modeling. Consistent with previous studies, this study also indicated significant associations between horizontal trust and self-rated health and between vertical (institutional) trust in the healthcare system and health. Further, significant sociodemographic determinants for health include younger age, male gender, marital status, high income, and high education.

Although the current study has inferential limitations for causal direction due to the cross-sectional study design, the interpretation could be made that the levels of trust in mass media may be able to influence the individual's health status. Enhancement of trust in mass media among the general population could be utilized to promote people's health.

Regarding causal pathways for how trust in mass media operates to influence health, the following mechanism can be considered: greater media trust may lead to higher use of mass media for health information; this in turn may lead to higher awareness of important health information and may result in better health-related decision-making and behavior. Alternatively, media trust could reflect higher credibility of public information on health issues, and may lead to greater dissemination of accurate health information, which may, in turn, lead to better health-related behavior. However, since there may be intermediate variables that underlie the relationship between media trust and health, further studies are needed to explore these causal mechanisms.

Mass media can have beneficial effects on people's health through conveying useful information related to health by various approaches, such as educational campaigns, series programs, and advertisements. In particular, mass media campaigns can have beneficial effects on public health, because mass media, particularly newspaper and television, can reach population-wide consumers throughout Asian countries. Given the widespread influence of mass media, well-designed mass media campaigns can have beneficial effects not only on health knowledge and attitudes, but also on health behaviors, with a potentially huge public health impact [25].

TV advertisements can increase public knowledge and awareness of the important symptoms of various diseases. For instance, TV delivery of information regarding the early warning symptoms of stroke increases the number of presentations to the emergency department during the early stages of stroke, providing increased opportunity to receive potentially life-saving thrombolytic therapies that are only indicated during the early stage [26,27]. A US study also showed that TV advertisements are the most frequently mentioned source of help among recent quitters of smoking [28]. Furthermore, a number of studies have shown that mass media campaigns enhance improvements in attitude toward healthy behavior, such as better diet, exercise, illegal drug prevention, safe sex, and smoking cessation [29-36]. The World Health Organization (WHO) reports on developing countries also support mass media interventions to increase the knowledge of HIV transmission and boost awareness of health providers [37].

Despite increased interest in obtaining health information by the public, a significant proportion of those diagnosed with a serious disease, such as cancer, report that they do not seek health information beyond that given by healthcare providers. One study, based on a national survey of American adults, demonstrated that compared with information-seeking groups, non-seeker patients showed low trust in mass media and paid less attention to health information in mass media [38]. Thus, trust in mass media is related to seeking behavior for health information and low trust may be associated with low levels of knowledge regarding important information relevant to their own health.

There are several strengths of our study. This may be one of the first studies to suggest a significant association between trust in mass media and health. Second, our results are based on the multilevel and multivariable model adjusted for potential confounders, such as demographic and socioeconomic factors. In evaluating the relationship between trust and well-being, these factors should be adjusted for to avoid confounding effects. Individuals with higher socioeconomic status may perceive their societies as being friendly and may have high trust in most public institutions, compared with those with a lower socioeconomic status [39]. Furthermore, socioeconomic status is related to health status [40]. Marital status is also associated with an individual's health and may be related to trust in public institutions [4]. The results based on the adjusted model are more reliable for estimating the association between trust and health.

Third, we assessed the potential association between sociodemographic factors and health after accounting for horizontal and vertical trust. The results of our study confirmed previous reports that found several factors for good health: including younger age, marital status, high income, high education, horizontal trust, and trust in the healthcare system [4,6,41]. In contrast, employment was not associated with health in our study.Thus, the typical 'healthy' Asians may be young, married, high-income, and highly educated men with a high trust in interpersonal relations as well as in the healthcare system and mass media.

Our study is based on the analysis of cross-sectional data and thus it has inferential limitations. It is possible that poor health leads to social isolation and distrust in any institutions due to psychosocial mechanisms. In addition, health and trust may reflect different facets of a common underlying psychological construct of general well-being. Alternatively, media trust might act as a surrogate marker for other types of output vertical trust, economic development or income equity in a country, or it might approximate the political systems, such as democracy, freedom of the press, and multi-ethnic cohesion. These parameters are known to be related to health status. Another limitation of our study was the use of the self-reported health satisfaction measure. It would have been more accurate to obtain more explicit self-reported health dimensions, such as those from the SF-36, although these data were not available in the AsiaBarometer Survey. Finally, our study has both cross-sectional causality problems and the absence of objective measures of physical health [42]. Future studies with a panel structure with individual fixed effects and more objective health measures, such as healthcare access or disability, are needed to mitigate the bias from omitting unobservable, personal, psychosocial characteristics, and to address measurement problems relating to self-reported health status [42].

In summary, this study is the first to analyze the relationship between high institutional trust in mass media and good health. These results indicate that individuals with high trust in mass media have better health. Mass media programs may contribute towards better health, especially among those people who have trust in mass media. Mass media may need to recognize the importance of their social role in terms of public health. Further research is necessary to determine the characteristics of high-quality mass media with high trust among the public.

## Competing interests

The authors declare that they have no competing interests.

## Authors' contributions

YT was involved in the analysis and interpretation of data, critical revision of the manuscript for important intellectual content, statistical analysis, and also had full access to all of the data in the study and takes responsibility for the integrity of the data and the accuracy of the data analysis. SF was involved in the acquisition, analysis, and interpretation of data and critical revision of the manuscript for important intellectual content. MJ was involved in interpretation of data and critical revision of the manuscript for important intellectual content. TI was responsible for the study concept and design, obtaining funds, administrative, technical, and material support, and study supervision, and was also involved in the acquisition, analysis, and interpretation of data and critical revision of the manuscript for important intellectual content.

## Pre-publication history

The pre-publication history for this paper can be accessed here:



## Supplementary Material

Additional file 1 **Appendix A and Appendix B** Click here for file
