# Supplementary material for: The relationship between trust in mass media and the healthcare system and individual health: evidence from the AsiaBarometer Survey
Source: BMC Med. 2009 Jan 22;7:4. doi: 10.1186/1741-7015-7-4 (PMC2655302; doi:10.1186/1741-7015-7-4)
Supplement: Additional file 1 — Appendix A and Appendix B [file 1741-7015-7-4-S1.doc]

**Appendix A: annual household income**

Categories of income groups included low-, mid-, and high-income groups, based on the income distribution of each country as follows:

1 = Low

2 = Mid

3 = High

The criterion used to assign these values is based on the sample to divide the samples into the three categories with the similar frequencies. Thus, we divide the samples of each country into the sub-samples with frequencies as close to 33% each as possible.

Year 2003

| Japan (Japanese yen) | | Group |
| --- | --- | --- |
| 1 | no income | 1 |
| 2 | less than 1 millions | 1 |
| 3 | 1-2 millions | 1 |
| 4 | 2-3 millions | 1 |
| 5 | 3-4 millions | 2 |
| 6 | 4-5 millions | 2 |
| 7 | 5-6 millions | 2 |
| 8 | 6-7 millions | 3 |
| 9 | 7-8 millions | 3 |
| 10 | 8-10 millions | 3 |
| 11 | 10 millions or more | 3 |
|  |  |  |
| South Korea (1000 won) | |  |
| 1 | Less Than 1200 | 1 |
| 2 | 1201-1800 | 1 |
| 3 | 1801-2400 | 1 |
| 4 | 2401-3000 | 1 |
| 5 | 3001-3600 | 2 |
| 6 | 3601-4200 | 2 |
| 7 | 4201-4800 | 3 |
| 8 | 4801-5400 | 3 |
| 9 | 5401-6000 | 3 |
| 10 | 6001-6600 | 3 |
| 11 | 6601-7200 | 3 |
| 12 | 7201-7800 | 3 |
| 13 | 7801-8400 | 3 |
| 14 | 8401 or more | 3 |
|  |  |  |
| China (yuan) | |  |
| 1 | Less Than 10000 | 1 |
| 2 | 10001-20000 | 1 |
| 3 | 20001-30000 | 2 |
| 4 | 30001-40000 | 2 |
| 5 | 40001-50000 | 3 |
| 6 | 50001-60000 | 3 |
| 7 | 60001-70000 | 3 |
| 8 | 70001-80000 | 3 |
| 9 | 80001-90000 | 3 |
| 10 | 90001-100000 | 3 |
| 11 | 100001-110000 | 3 |
| 12 | 110001 or more | 3 |
|  |  |  |
| Malaysia (RM) | |  |
| 1 | Below 5000 | 1 |
| 2 | 5001-10000 | 1 |
| 3 | 10001-15000 | 1 |
| 4 | 15001-20000 | 2 |
| 5 | 20001-30000 | 2 |
| 6 | 30001-50000 | 3 |
| 7 | 50001-80000 | 3 |
| 8 | 80001-100000 | 3 |
| 9 | 100001-150000 | 3 |
| 10 | 150001-300000 | 3 |
| 11 | 300001-500000 | 3 |
| 12 | more than 500000 | 3 |
|  |  |  |
| Thailand (bhat) | |  |
| 1 | less than 10000 | 1 |
| 2 | 10000-15000 | 2 |
| 3 | 15001-20000 | 2 |
| 4 | 20001-25000 | 3 |
| 5 | 25001-30000 | 3 |
| 6 | 30001-35000 | 3 |
| 7 | 35001-40000 | 3 |
| 8 | 40001-45000 | 3 |
| 9 | 45001-50000 | 3 |
| 10 | 50001-55000 | 3 |
| 11 | 55001-60000 | 3 |
| 12 | 60001-65000 | 3 |
| 13 | 65001-70000 | 3 |
| 14 | more than 70000 | 3 |
|  |  |  |
| Vietnam (dong) | |  |
| 1 | 12 millions or below | 1 |
| 2 | 12-24 millions | 2 |
| 3 | 24-36 millions | 3 |
| 4 | 36-48 millions | 3 |
| 5 | 48-60 millions | 3 |
| 6 | 60-72 millions | 3 |
| 7 | 72-84 millions | 3 |
| 8 | 84-96 millions | 3 |
| 9 | 96-108 millions | 3 |
| 10 | 108-120 millions | 3 |
| 11 | 120-132 millions | 3 |
| 12 | 132-144 millions | 3 |
| 13 | 144-156 millions | 3 |
| 14 | 156-168 millions | 3 |
| 15 | 168-180 millions | 3 |
| 16 | 180 millions or more | 3 |
|  |  |  |
| Myanmar (kyat) | |  |
| 1 | Below 100000 | 1 |
| 2 | 100001-250000 | 1 |
| 3 | 250001-350000 | 1 |
| 4 | 350001-450000 | 2 |
| 5 | 450001-600000 | 2 |
| 6 | 600001-850000 | 3 |
| 7 | 850001-1000000 | 3 |
| 8 | 1000001-1500000 | 3 |
| 9 | 1500001-2500000 | 3 |
| 10 | 2500001-3500000 | 3 |
| 11 | 3500001-4500000 | 3 |
| 12 | 4500001-5500000 | 3 |
| 13 | 5500001-6500000 | 3 |
| 14 | 6500001-9500000 | 3 |
| 15 | 9500001 or more | 3 |
|  |  |  |
| India (Indian Rupees) | |  |
| 1 | 4000 or less | 1 |
| 2 | 4001-5000 | 1 |
| 3 | 5001-6000 | 2 |
| 4 | 6001-7000 | 2 |
| 5 | 7001-8000 | 2 |
| 6 | 8001-9000 | 3 |
| 7 | 9001-10000 | 3 |
| 8 | 10001-11000 | 3 |
| 9 | 11001-12000 | 3 |
| 10 | 12001-13000 | 3 |
| 11 | 13001-14000 | 3 |
| 12 | 14001-15000 | 3 |
| 13 | 15001-16000 | 3 |
| 14 | 16001-20000 | 3 |
| 15 | 20001 and above | 3 |
|  |  |  |
| Sri Lanka (Sri Lanka Rupees) | |  |
| 1 | 2000 or less | 1 |
| 2 | 2001-3000 | 1 |
| 3 | 3001-4000 | 1 |
| 4 | 4001-5000 | 1 |
| 5 | 5001-6000 | 1 |
| 6 | 6001-7000 | 1 |
| 7 | 7001-8000 | 2 |
| 8 | 8001-9000 | 2 |
| 9 | 9001-10000 | 2 |
| 10 | 10001-11000 | 2 |
| 11 | 11001-12000 | 2 |
| 12 | 12001-13000 | 2 |
| 13 | 13001-14000 | 3 |
| 14 | 14001-15000 | 3 |
| 15 | 15001-16000 | 3 |
| 16 | 16001-17000 | 3 |
| 17 | 17001-18000 | 3 |
| 18 | 18001-19000 | 3 |
| 19 | 19001-20000 | 3 |
| 20 | more than 20000 | 3 |
|  |  |  |
| Uzbekistan (1000 sum) | |  |
| 1 | 20 or less | 1 |
| 2 | 21-40 | 1 |
| 3 | 41-60 | 1 |
| 4 | 61-80 | 1 |
| 5 | 81-100 | 1 |
| 6 | 101-150 | 1 |
| 7 | 151-200 | 1 |
| 8 | 201-250 | 1 |
| 9 | 251-300 | 1 |
| 10 | 301-350 | 1 |
| 11 | 351-400 | 1 |
| 12 | 401-500 | 2 |
| 13 | 501-600 | 2 |
| 14 | 601-700 | 2 |
| 15 | 701-800 | 2 |
| 16 | 801-900 | 2 |
| 17 | 901-1000 | 3 |
| 18 | 1001-2000 | 3 |
| 19 | 2001-3000 | 3 |
| 20 | 3001-4000 | 3 |
| 21 | 4001-5000 | 3 |
| 22 | 5001-6000 | 3 |
| 23 | 6001-7000 | 3 |
| 24 | more than 7000 | 3 |

Year 2004

| Cambodia (Cambodian Riel) | | Group |
| --- | --- | --- |
| 1 | Under 2.4 million | 1 |
| 2 | 2.4 to 4.8 million | 1 |
| 3 | 4.8 to 7.2 million | 1 |
| 4 | 7.2 to 9.6 million | 1 |
| 5 | 9.6 to 12.0 million | 2 |
| 6 | 12.0 to 14.4 million | 2 |
| 7 | 14.4 to 16.8 million | 3 |
| 8 | 16.8 to 19.2 million | 3 |
| 9 | 19.2 to 28.8 million | 3 |
| 10 | 28.8 to 38.4 million | 3 |
| 11 | 38.4 to 48.0 million | 3 |
| 12 | more than 48.0 million | 3 |
|  |  |  |
| Indonesia (Indonesian Rupiah) | |  |
| 1 | Under 6 million | 1 |
| 2 | 6 to 7.2 million | 1 |
| 3 | 7.2 to 8.4 million | 2 |
| 4 | 8.4 to 9.6 million | 2 |
| 5 | 9.6 to 12 million | 3 |
| 6 | 12 to 18 million | 3 |
| 7 | 18 to 24 million | 3 |
| 8 | 24 to 36 million | 3 |
| 9 | more than 36 million | 3 |
|  |  |  |
| Japan (Japanese Yen) | |  |
| 1 | 2 million or less | 1 |
| 2 | 2 to 3 million | 1 |
| 3 | 3 to 4 million | 1 |
| 4 | 4 to 5 million | 1 |
| 5 | 5 to 6 million | 2 |
| 6 | 6 to 7 million | 2 |
| 7 | 7 to 8 million | 2 |
| 8 | 8 to 9 million | 3 |
| 9 | 9 to 10 million | 3 |
| 10 | 10 to 11 million | 3 |
| 11 | 11 to 12 million | 3 |
| 12 | 12 to 13 million | 3 |
| 13 | 13 to 14 million | 3 |
| 14 | 14 to 15 million | 3 |
| 15 | 15 to 16 million | 3 |
| 16 | 16 to 17 million | 3 |
| 17 | 17 to 18 million | 3 |
| 18 | 18 to 19 million | 3 |
| 19 | 19 to 20 million | 3 |
| 20 | more than 20 million | 3 |
|  |  |  |
| Myanmar (Myanma Kyat) | |  |
| 1 | Below 100,000 | 1 |
| 2 | 100,001-250,000 | 1 |
| 3 | 250,001-350,000 | 1 |
| 4 | 350,001-450,000 | 1 |
| 5 | 450,001-600,000 | 2 |
| 6 | 600,001-850,000 | 3 |
| 7 | 850,001-1,000,000 | 3 |
| 8 | 1,000,001-1,500,000 | 3 |
| 9 | 1,500,001-2,500,000 | 3 |
| 10 | 2,500,001-3,500,000 | 3 |
| 11 | 3,500,001-4,500,000 | 3 |
| 12 | 4,500,001-5,500,000 | 3 |
| 13 | 5,500,001-6,500,000 | 3 |
| 14 | 6,500,001-9,500,000 | 3 |
| 15 | over 9,500,001 | 3 |
|  |  |  |
| Philippines (Filipino Peso) | |  |
| 1 | Below 3,000 | 1 |
| 2 | 3,001-5,000 | 1 |
| 3 | 5,001-6,000 | 1 |
| 4 | 6,001-7,000 | 1 |
| 5 | 7,001-8,000 | 1 |
| 6 | 8,001-9,000 | 1 |
| 7 | 9,001-10,000 | 1 |
| 8 | 10,001-15,000 | 1 |
| 9 | 15,001-20,000 | 1 |
| 10 | 20,001-25,000 | 1 |
| 11 | 25,001-30,000 | 1 |
| 12 | 30,001-35,000 | 1 |
| 13 | 35,001-40,000 | 1 |
| 14 | 40,001-45,000 | 1 |
| 15 | 45,001-50,000 | 1 |
| 16 | 50,001-60,000 | 1 |
| 17 | 60,001-70,000 | 1 |
| 18 | 70,001-80,000 | 2 |
| 19 | 80,001-90,000 | 2 |
| 20 | 90,001-100,000 | 2 |
| 21 | 100,001 or more | 3 |
|  |  |  |
| Thailand (Thai Baht) | |  |
| 1 | Under 5,000 | 1 |
| 2 | 5,000-5,999 | 2 |
| 3 | 6,000-7,999 | 2 |
| 4 | 8,000-9,999 | 2 |
| 5 | 10,000-12,499 | 3 |
| 6 | 12,500-14,999 | 3 |
| 7 | 15,000-17,499 | 3 |
| 8 | 17,500-19,999 | 3 |
| 9 | 20,000-24,999 | 3 |
| 10 | 25,000-29,999 | 3 |
| 11 | 30,000-34,999 | 3 |
| 12 | 35,000-39,999 | 3 |
| 13 | 40,000-44,999 | 3 |
| 14 | 45,000-49,999 | 3 |
| 15 | 50,000-54,999 | 3 |
| 16 | 55,000-59,999 | 3 |
| 17 | over 60,000 | 3 |
|  |  |  |
| Vietnam (Vietnamese Dong) | |  |
| 1 | Under 12 million | 1 |
| 2 | 12-24 million | 1 |
| 3 | 24-36 million | 2 |
| 4 | 36-48 million | 3 |
| 5 | 48-60 million | 3 |
| 6 | 60-72 million | 3 |
| 7 | 72-84 million | 3 |
| 8 | 84-96 million | 3 |
| 9 | 96-108 million | 3 |
| 10 | 108-120 million | 3 |
| 11 | 120-132 million | 3 |
| 12 | 132-144 million | 3 |
| 13 | 144-156 million | 3 |
| 14 | 156-168 million | 3 |
| 15 | 168-180 million | 3 |
| 16 | over 180 million | 3 |
|  |  |  |
| Malaysia (Malaysian Ringgit) | |  |
| 1 | Below 5,000 | 1 |
| 2 | 5,001-10,000 | 1 |
| 3 | 10,001-15,000 | 2 |
| 4 | 15,001-20,000 | 2 |
| 5 | 20,000-30,000 | 2 |
| 6 | 30,001-50,000 | 3 |
| 7 | 50,001-80,000 | 3 |
| 8 | 80,001-100,000 | 3 |
| 9 | 100,000-150,000 | 3 |
| 10 | 150,000-300,000 | 3 |
| 11 | 300,001-500,000 | 3 |
| 12 | over 500,001 | 3 |
|  |  |  |
| Singapore (Singaporean Dollars) | |  |
| 1 | No income | 1 |
| 2 | Below 1,000 | 1 |
| 3 | 1,001-2,000 | 1 |
| 4 | 2,001-3,000 | 2 |
| 5 | 3,001-4,000 | 2 |
| 6 | 4,001-5,000 | 3 |
| 7 | 5,001-6,000 | 3 |
| 8 | 6,001-7,000 | 3 |
| 9 | 7,001-8,000 | 3 |
| 10 | 8,001-9,000 | 3 |
| 11 | 9,001-10,000 | 3 |
| 12 | over 10,001 | 3 |
|  |  |  |
| Korea (1000 Won) | |  |
| 1 | 20 or less (million won) | 1 |
| 2 | 20-30 | 1 |
| 3 | 30-40 | 2 |
| 4 | 40-50 | 3 |
| 5 | 50-60 | 3 |
| 6 | 60-70 | 3 |
| 7 | 70-80 | 3 |
| 8 | 80-90 | 3 |
| 9 | 90-100 | 3 |
| 10 | 100-110 | 3 |
| 11 | 110-120 | 3 |
| 12 | 120-130 | 3 |
| 13 | 130-140 | 3 |
| 14 | 140-150 | 3 |
| 15 | 150-160 | 3 |
| 16 | 160-170 | 3 |
| 17 | 170-180 | 3 |
| 18 | 180-190 | 3 |
| 19 | 190-200 | 3 |
| 20 | more than 200 | 3 |
|  |  |  |
| Laos (Lao Kip) | |  |
| 1 | Below 60,000 | 1 |
| 2 | 60,001-100,000 | 1 |
| 3 | 100,001-200,000 | 1 |
| 4 | 200,001-300,000 | 1 |
| 5 | 300,001-500,000 | 1 |
| 6 | 500,001-800,000 | 1 |
| 7 | 800,000-1,000,000 | 2 |
| 8 | 1,000,001-1,500,000 | 3 |
| 9 | 1,500,001-2,000,000 | 3 |
| 10 | 2,000,001-3,000,000 | 3 |
| 11 | 3,000,001-5,000,000 | 3 |
| 12 | 5,000,001-8,000,000 | 3 |
| 13 | 8,000,001-10,000,000 | 3 |
| 14 | 10,000,001-15,000,000 | 3 |
| 15 | 15,000,001-20,000,000 | 3 |
| 16 | Over 20,000,001 | 3 |
|  |  |  |
| China (Chinese Yuan) | |  |
| 1 | 20000 or lower | 1 |
| 2 | 20001-30000 | 2 |
| 3 | 30001-40000 | 2 |
| 4 | 40001-50000 | 3 |
| 5 | 50001-60000 | 3 |
| 6 | 60001-70000 | 3 |
| 7 | 70001-80000 | 3 |
| 8 | 80001-90000 | 3 |
| 9 | 90001-100000 | 3 |
| 10 | 100001-110000 | 3 |
| 11 | 110001-120000 | 3 |
| 12 | 120001-130000 | 3 |
| 13 | 130001-140000 | 3 |
| 14 | 140001-150000 | 3 |
| 15 | 150001-160000 | 3 |
| 16 | 160001-170000 | 3 |
| 17 | 170001-180000 | 3 |
| 18 | 180001-190000 | 3 |
| 19 | 190001-200000 | 3 |
| 20 | 200001 or more | 3 |
|  |  |  |
| Brunei (Brunei Dollar) | |  |
| 1 | Below B$15,000 | 1 |
| 2 | B$15,001-B$30,000 | 2 |
| 3 | B$30,001-B$50,000 | 2 |
| 4 | B$50,001-B$80,000 | 3 |
| 5 | B$80,001-B$100,000 | 3 |
| 6 | B$100,001-B$150,000 | 3 |
| 7 | B$150,001-B$300,000 | 3 |
| 8 | B$300,001-B$500,000 | 3 |
| 9 | More than B$500,000 | 3 |
| 10 | More than B$500,000 | 3 |

Year 2005

Afghanistan: same as the above.

| Bangladesh (Taka) | |  |
| --- | --- | --- |
| 1 | 999 or less | 1 |
| 2 | 1000 – 1999 | 1 |
| 3 | 2000 – 2999 | 1 |
| 4 | 3000 – 3999 | 2 |
| 5 | 4000 – 4999 | 2 |
| 6 | 5000 – 5999 | 2 |
| 7 | 6000 – 6999 | 3 |
| 8 | 7000 – 7999 | 3 |
| 9 | 8000 – 8999 | 3 |
| 10 | 9000 – 9999 | 3 |
| 11 | 10000 – 10999 | 3 |
| 12 | 11000 – 11999 | 3 |
| 13 | 12000 – 12999 | 3 |
| 14 | 13000 – 14999 | 3 |
| 15 | 15000 – 16999 | 3 |
| 16 | 17000 – 19999 | 3 |
| 17 | 20000 – 24999 | 3 |
| 18 | 25000 – 34999 | 3 |
| 19 | 35000 – 49999 | 3 |
| 20 | More than 50000 | 3 |
|  |  |  |
| Bhutan (Rs) | |  |
| 1 | Up to 2500 | 1 |
| 2 | 2501 – 5000 | 1 |
| 3 | 5001 – 7500 | 1 |
| 4 | 7501 – 10000 | 1 |
| 5 | 10001 – 12500 | 1 |
| 6 | 12501 – 15000 | 2 |
| 7 | 15001 – 17500 | 2 |
| 8 | 17501 – 20000 | 3 |
| 9 | 20001 – 22500 | 3 |
| 10 | 22501 – 25000 | 3 |
| 11 | 25001 above | 3 |
|  |  |  |
| India (Rs) | |  |
| 1 | Up to 2500 | 1 |
| 2 | 2501 – 5000 | 1 |
| 3 | 5001 – 7500 | 1 |
| 4 | 7501 – 10000 | 2 |
| 5 | 10001 – 12500 | 2 |
| 6 | 12501 – 15000 | 3 |
| 7 | 15001 – 17500 | 3 |
| 8 | 17501 – 20000 | 3 |
| 9 | 20001 – 22500 | 3 |
| 10 | 22501 – 25000 | 3 |
| 11 | 25001 above | 3 |
|  |  |  |
| Kazakhstan (Tenge) | |  |
| 1 | 10000 and less | 1 |
| 2 | 10001 – 15000 | 1 |
| 3 | 15001 – 20000 | 1 |
| 4 | 20001 – 25000 | 2 |
| 5 | 25001 – 30000 | 2 |
| 6 | 30001 – 35000 | 2 |
| 7 | 35001 – 40000 | 2 |
| 8 | 40001 – 45000 | 3 |
| 9 | 45001 – 50000 | 3 |
| 10 | 50001 – 55000 | 3 |
| 11 | 55001 – 60000 | 3 |
| 12 | 60001 – 65000 | 3 |
| 13 | 65001 – 70000 | 3 |
| 14 | 70001 – 75000 | 3 |
| 15 | 75001 – 80000 | 3 |
| 16 | 80001 – 85000 | 3 |
| 17 | 85001 – 90000 | 3 |
| 18 | 90001 – 95000 | 3 |
| 19 | 95001 –100000 | 3 |
| 20 | 100001 and more | 3 |
|  |  |  |
| Kyrgyzstan (Com) | |  |
| 1 | Less than 10000 | 1 |
| 2 | 10001 – 15000 | 1 |
| 3 | 15001 – 20000 | 1 |
| 4 | 20001 – 25000 | 1 |
| 5 | 25001 – 30000 | 1 |
| 6 | 30001 – 35000 | 1 |
| 7 | 35001 – 40000 | 1 |
| 8 | 40001 – 45000 | 1 |
| 9 | 45001 – 50000 | 1 |
| 10 | 50001 – 55000 | 1 |
| 11 | 55001 – 60000 | 1 |
| 12 | 60001 – 65000 | 1 |
| 13 | 65001 – 70000 | 2 |
| 14 | 70001 – 75000 | 2 |
| 15 | 75001 – 80000 | 2 |
| 16 | 80001 – 85000 | 2 |
| 17 | 85001 – 90000 | 2 |
| 18 | 90001 – 95000 | 2 |
| 19 | 95001 – 100000 | 2 |
| 20 | 100001 and more | 3 |
|  |  |  |
| Maldives (Rs) | |  |
| 1 | Up to 2500 | 1 |
| 2 | 2501 – 5000 | 1 |
| 3 | 5001 – 7500 | 1 |
| 4 | 7501 – 10000 | 1 |
| 5 | 10001 – 12500 | 2 |
| 6 | 12501 – 15000 | 3 |
| 7 | 15001 – 17500 | 3 |
| 8 | 17501 – 20000 | 3 |
| 9 | 20001 – 22500 | 3 |
| 10 | 22501 – 25000 | 3 |
| 11 | 25001 and above | 3 |
|  |  |  |
| Mongolia (Ts) | |  |
| 1 | 240 or less | 1 |
| 2 | 240-600 | 1 |
| 3 | 600-1200 | 2 |
| 4 | 1200-1800 | 3 |
| 5 | 1800-3000 | 3 |
| 6 | 3000-4200 | 3 |
| 7 | more than 4200 | 3 |
|  |  |  |
| Nepal (Rs) | |  |
| 1 | Up to 2500 | 1 |
| 2 | 2501 – 5000 | 1 |
| 3 | 5001 – 7500 | 1 |
| 4 | 7501 – 10000 | 2 |
| 5 | 10001 – 12500 | 2 |
| 6 | 12501 – 15000 | 3 |
| 7 | 15001 – 17500 | 3 |
| 8 | 17501 – 20000 | 3 |
| 9 | 20001 – 22500 | 3 |
| 10 | 22501 – 25000 | 3 |
| 11 | 25001 or above | 3 |
|  |  |  |
| Pakistan (Rs) | |  |
| 1 | Less than 36000 | 1 |
| 2 | 36001 – 50000 | 2 |
| 3 | 50001 – 75000 | 2 |
| 4 | 75001 – 100000 | 3 |
| 5 | 100001 – 150000 | 3 |
| 6 | 150001 – 200000 | 3 |
| 7 | 200001 – 300000 | 3 |
| 8 | 300001 – 400000 | 3 |
| 9 | 400001 – 500000 | 3 |
| 10 | More than 500000 | 3 |
|  |  |  |
| Sri Lanka (Rs) | |  |
| 1 | Up to 2500 | 1 |
| 2 | 2501 – 5000 | 1 |
| 3 | 5001 – 7500 | 1 |
| 4 | 7501 – 10000 | 1 |
| 5 | 10001 – 12500 | 2 |
| 6 | 12501 – 15000 | 2 |
| 7 | 15001 – 17500 | 3 |
| 8 | 17501 – 20000 | 3 |
| 9 | 20001 – 22500 | 3 |
| 10 | 22501 – 25000 | 3 |
| 11 | 25001 above | 3 |
|  |  |  |
| Tajikistan (TJR) | |  |
| 1 | Less than 200 | 1 |
| 2 | 201 – 250 | 1 |
| 3 | 251 – 300 | 1 |
| 4 | 301 – 350 | 1 |
| 5 | 351 – 400 | 1 |
| 6 | 401 – 450 | 1 |
| 7 | 451 – 500 | 1 |
| 8 | 501 – 550 | 1 |
| 9 | 551 – 600 | 1 |
| 10 | 601 – 650 | 1 |
| 11 | 651 – 700 | 1 |
| 12 | 701 – 750 | 1 |
| 13 | 751 – 800 | 1 |
| 14 | 801 – 850 | 1 |
| 15 | 851 – 900 | 1 |
| 16 | 901 – 950 | 1 |
| 17 | 951 – 1000 | 1 |
| 18 | 1001 – 1500 | 1 |
| 19 | 1501 – 2000 | 2 |
| 20 | More than 2000 | 3 |
|  |  |  |
| Turkmenistan (Million manta) | |  |
| 1 | Less than 10 | 1 |
| 2 | 11 – 15 | 1 |
| 3 | 16 – 20 | 1 |
| 4 | 21 – 25 | 2 |
| 5 | 26 – 30 | 2 |
| 6 | 31 – 35 | 3 |
| 7 | 36 – 40 | 3 |
| 8 | 41 – 45 | 3 |
| 9 | 46 – 50 | 3 |
| 10 | 51 – 55 | 3 |
| 11 | 56 – 60 | 3 |
| 12 | 61 – 65 | 3 |
| 13 | 66 – 70 | 3 |
| 14 | 71 – 75 | 3 |
| 15 | 76 – 80 | 3 |
| 16 | 81 – 85 | 3 |
| 17 | 86 – 90 | 3 |
| 18 | 91 – 95 | 3 |
| 19 | 96 – 100 | 3 |
| 20 | More than 100 | 3 |
|  |  |  |
| Uzbekistan (Uzbek sums) | |  |
| 1 | 100000 or lower | 1 |
| 2 | 101 to 150 thousand | 1 |
| 3 | 151 to 200 thousand | 1 |
| 4 | 201 to 250 thousand | 1 |
| 5 | 251 to 300 thousand | 1 |
| 6 | 301 to 400 thousand | 1 |
| 7 | 401 to 500 thousand | 1 |
| 8 | 501 to 600 thousand | 1 |
| 9 | 601 to 700 thousand | 1 |
| 10 | 701 to 800 thousand | 1 |
| 11 | 801 to 900 thousand | 1 |
| 12 | 901 to 999 thousand | 2 |
| 13 | 1 to 2 million | 2 |
| 14 | 2 to 3 million | 3 |
| 15 | 3 to 4 million | 3 |
| 16 | 4 to 5 million | 3 |
| 17 | 5 to 6 million | 3 |
| 18 | 6 to 7 million | 3 |
| 19 | 7 to 8 million | 3 |
| 20 | More than 8 million | 3 |

Year 2006

| China (Chinese Yuan) | |  |
| --- | --- | --- |
| 1 | Less than 20000 | 1 |
| 2 | 20000 to less than 30000 | 2 |
| 3 | 30000 to less than 40000 | 3 |
| 4 | 40000 to less than 50000 | 3 |
| 5 | 50000 to less than 60000 | 3 |
| 6 | 60000 to less than 70000 | 3 |
| 7 | 70000 to less than 80000 | 3 |
| 8 | 80000 to less than 90000 | 3 |
| 9 | 90000 to less than 100000 | 3 |
| 10 | 100000 to less than 110000 | 3 |
| 11 | 110000 to less than 120000 | 3 |
| 12 | 120000 to less than 130000 | 3 |
| 13 | 130000 to less than 140000 | 3 |
| 14 | 140000 to less than 150000 | 3 |
| 15 | 150000 to less than 160000 | 3 |
| 16 | 160000 to less than 170000 | 3 |
| 17 | 170000 to less than 180000 | 3 |
| 18 | 180000 to less than 190000 | 3 |
| 19 | 190000 to less than 200000 | 3 |
| 20 | 200000 or more | 3 |
|  |  |  |
| Hong Kong (Hong Kong dollars) | |  |
| 1 | Less than 50000 | 1 |
| 2 | 50000 to less than 70000 | 1 |
| 3 | 70000 to less than 90000 | 1 |
| 4 | 90000 to less than 110000 | 1 |
| 5 | 110000 to less than 130000 | 1 |
| 6 | 130000 to less than 150000 | 2 |
| 7 | 150000 to less than 180000 | 2 |
| 8 | 180000 to less than 210000 | 2 |
| 9 | 210000 to less than 240000 | 2 |
| 10 | 240000 to less than 270000 | 3 |
| 11 | 270000 to less than 300000 | 3 |
| 12 | 300000 to less than 350000 | 3 |
| 13 | 350000 to less than 400000 | 3 |
| 14 | 400000 to less than 450000 | 3 |
| 15 | 450000 to less than 500000 | 3 |
| 16 | 500000 to less than 600000 | 3 |
| 17 | 600000 to less than 700000 | 3 |
| 18 | 700000 to less than 800000 | 3 |
| 19 | 800000 to less than 900000 | 3 |
| 20 | 1 million or more | 3 |
|  |  |  |
| Japan (Japanese yen) | |  |
| 1 | less than 2 million | 1 |
| 2 | 2 to 3 million | 1 |
| 3 | 3 to 4 million | 1 |
| 4 | 4 to 5 million | 2 |
| 5 | 5 to 6 million | 2 |
| 6 | 6 to 7 million | 3 |
| 7 | 7 to 8 million | 3 |
| 8 | 8 to 9 million | 3 |
| 9 | 9 to 10 million | 3 |
| 10 | 10 to 11 million | 3 |
| 11 | 11to 12 million | 3 |
| 12 | 12 to 13 million | 3 |
| 13 | 13 to 14 million | 3 |
| 14 | 14 to 15 million | 3 |
| 15 | 15 to 16 million | 3 |
| 16 | 16 to 17 million | 3 |
| 17 | 17 to 18 million | 3 |
| 18 | 18 to 19 million | 3 |
| 19 | 19 to 20 million | 3 |
| 20 | more than 20 million | 3 |
|  |  |  |
| South Korea (South Korea won) | |  |
| 1 | Less than 20M | 1 |
| 2 | 20M to less than 30M | 2 |
| 3 | 30M to less than 40M | 2 |
| 4 | 40M to less than 50M | 3 |
| 5 | 50M to less than 60M | 3 |
| 6 | 60M to less than 70M | 3 |
| 7 | 70m to less than 80M | 3 |
| 8 | 80M to less than 90M | 3 |
| 9 | 90M to less than 100M | 3 |
| 10 | 100M to less than 110M | 3 |
| 11 | 110M to less than 120M | 3 |
| 12 | 120M to less than 130M | 3 |
| 13 | 130M to less than 140M | 3 |
| 14 | 140M to less than 150M | 3 |
| 15 | 150M to less than 160M | 3 |
| 16 | 160M to less than 170M | 3 |
| 17 | 170M to less than 180M | 3 |
| 18 | 180M to less than 190M | 3 |
| 19 | 190M to less than 200M | 3 |
| 20 | 200M or more | 3 |
|  |  |  |
| Singapore (Singapore dollars) | |  |
| 1 | No income | 1 |
| 2 | 1000 or below | 1 |
| 3 | 1001-2000 | 1 |
| 4 | 2001-3000 | 2 |
| 5 | 3001-4000 | 2 |
| 6 | 4001-5000 | 3 |
| 7 | 5001-6000 | 3 |
| 8 | 6001-7000 | 3 |
| 9 | 7001-8000 | 3 |
| 10 | 8001-9000 | 3 |
| 11 | 9001-10000 | 3 |
| 12 | more than 10000 | 3 |
|  |  |  |
| Taiwan (Taiwan yuan) | |  |
| 1 | Less than 30000 | 1 |
| 2 | 30000-39999 | 1 |
| 3 | 40000-49999 | 1 |
| 4 | 50000-54999 | 1 |
| 5 | 55000-59999 | 2 |
| 6 | 60000-64999 | 2 |
| 7 | 65000-69999 | 2 |
| 8 | 70000-74999 | 2 |
| 9 | 75000-79999 | 2 |
| 10 | 80000-84999 | 3 |
| 11 | 85000-89999 | 3 |
| 12 | 90000-94999 | 3 |
| 13 | 95000-99999 | 3 |
| 14 | 100000-109999 | 3 |
| 15 | 110000-119999 | 3 |
| 16 | 120000-129999 | 3 |
| 17 | 130000-149999 | 3 |
| 18 | 150000-199999 | 3 |
| 19 | 200000 or more | 3 |
|  |  |  |
| Vietnam (Vietnam dong) | |  |
| 1 | 12000000 or less | 1 |
| 2 | 12000001-24000000 | 1 |
| 3 | 24000001-36000000 | 2 |
| 4 | 36000001-48000000 | 2 |
| 5 | 48000001-60000000 | 3 |
| 6 | 60000001-72000000 | 3 |
| 7 | 72000001-84000000 | 3 |
| 8 | 84000001-96000000 | 3 |
| 9 | 96000001-108000000 | 3 |
| 10 | 10800001-120000000 | 3 |
| 11 | 12000001-132000000 | 3 |
| 12 | 13200001-144000000 | 3 |
| 13 | 14400001-156000000 | 3 |
| 14 | 15600001-168000000 | 3 |
| 15 | 16800001-180000000 | 3 |
| 16 | More than 180000000 | 3 |

**Appendix B: educational attainment**

Categories of educational attainment included the low-, mid-, and high-education groups, based on the distribution of educational attainment of each country:

Data in 2003-2005, all societies are coded as follows:

1 No formal education

2 Elementary school/junior high school/middle school

3 High school

4 High-school-level vocational-technical school

5 Professional school/technical school

6 University/graduate school

9 Don’t know

These codes are collapsed to the following:

1 No formal education: 1

2 Elementary school/junior high school/middle school: 1

3 High school: 2

4 High-school-level vocational-technical school: 2

5 Professional school/technical school: 3

6 University/graduate school: 3

1 = Low-education

2 = Mid-education

3 = High-education

Categories are different for each society in data of 2006 and the following reclassifications are used.

China

1 Primary school or below: 1

2 Junior high school/Middle school: 1

3 High school/Vocational school: 2

4 College school: 3

5 University: 3

6 Graduate school or above: 3

Hong Kong

1 No formal education: 1

2 Primary school: 1

3 Lower secondary school: 1

4 Senior secondary school: 2

5 Matriculation: 3

6 College/University or above: 3

Japan

1 Primary school/Junior high school: 1

2 High school: 2

3 Technical school: 3

4 College: 3

5 University/Graduate school: 3

South Korea

1 Elementary school: 1

2 Middle school: 1

3 High school: 2

4 College/University: 3

5 Graduate school or above: 3

Singapore

1 No formal education: 1

2 Primary school: 1

3 Secondary school/GCE O level: 1

4 Post secondary school: 2

5 GCE A/Diploma: 2

6 University/Graduate school: 3

Taiwan

1 No formal education: 1

2 Elementary school: 1

3 Junior high school/Middle school: 1

4 High school/Vocational school: 2

5 College school: 3

6 University/Graduate school: 3

Vietnam

1 No formal education: 1

2 Grade 1-5: 1

3 Grade 6-9: 1

4 Grade 10-12: 2

5 Studying in college: 3

6 Graduated college: 3

7 Studying in university: 3

8 Graduated university: 3

9 Post-university: 3
